# Structural insights into transition-metal–amino­diphosphine (PNP) com­plexes bearing [*M*Cl_*n*_(PNP)_2_] (*M* = Co, Ru, Cr or Mo; *n* = 1 or 2) cores in the solid state

**DOI:** 10.1107/S2053229625009519

**Published:** 2025-11-10

**Authors:** Sizwe J. Zamisa, Adesola A. Adeleke, Dunesha Naicker, Holger B. Friedrich, Bernard Omondi

**Affiliations:** a School of Agriculture and Science, Discipline of Chemistry, University of KwaZulu-Natal, Private Bag X54001, Westville, Durban, 4000, South Africa; University of the Witwatersrand, South Africa

**Keywords:** cobalt amino­diphosphine com­plexes, crystal structure, CSD survey, noncovalent inter­actions

## Abstract

The mol­ecular structures of two novel cobalt amino­diphosphine (PNP) com­plexes (**1** and **2**) are reported, namely, bis­[bis­(di­phenyl­phosphan­yl)(pent­yl)amine-κ^2^*P*,*P*′]chlorido­cobalt(III) di-μ-chlorido-bis­[di­chlorido­cobalt(II)], [CoCl(C_29_H_31_NP_2_)_2_][Co_2_Cl_6_], and bis­[bis­(di­phenyl­phosphan­yl)(propan-2-yl)amine-κ^2^*P*,*P*′]chlorido­cobalt(III) di-μ-chlorido-bis­[di­chlorido­cobalt(II)], [CoCl(C_27_H_27_NP_2_)_2_][Co_2_Cl_6_], featuring variation in the N-atom substituent, *i.e.n*-pentyl in com­plex **1** and isopropyl in com­plex **2**. The asymmetric unit of com­plex **1** contains a five-coordinated cationic [CoCl{κ^2^-*P*,*P*-(*N*)-C_5_H_11_}_2_]^2+^ species and a [Co_2_(μ_2_-Cl)_2_Cl_4_]^2−^ anion, while com­plex **2** includes a five-coordinated cation [CoCl{κ^2^-*P*,*P*-(*N*)-C_3_H_7_}_2_], half a [Co_2_(μ_2_-Cl)_2_Cl_4_]^2−^ anion, and disordered diethyl ether and di­chloro­methane solvent mol­ecules. The impact of ligand-induced strain, particularly due to the small bite angles of the PNP amino­diphosphine ligands, was examined in the context of geometric constraints and their influence on stability and reactivity. A Cambridge Structural Database (CSD) survey, along with a noncovalent inter­action (NCI) analysis of the analogous [TMCl_*n*_(PNP)_2_] (where TM = transition metal and *n* = 1 or 2) core, revealed an inverse correlation between P—TM—P bite angles and N⋯TM contact distances. This correlation is attributed to the influence of the van der Waals radius of the metal: smaller metals allow wider bite angles and stronger N⋯TM contacts, whereas larger metals favour narrower bite angles and longer N⋯TM distances. NCI analysis indicated significant steric repulsion at the TM⋯N contacts, reflecting strain imposed by ligand geometry. A com­parison of sign(λ_2_)ρ eigenvalues suggests that Mo—P bonds exhibit weaker attractive inter­actions relative to Co—P and Ru—P bonds, with Cr—P bonds being the weakest. These findings provide po­inters to structural and electronic factors governing coordination in PNP-ligated transition-metal com­plexes, offering rational design and catalytic and material applications.

## Abstract 2

The mol­ecular structures of two novel cobalt amino­diphosphine (PNP) com­plexes are reported, featuring variation in the N-atom substituent. A Cambridge Structural Database (CSD) survey, along with a noncovalent inter­action (NCI) analysis of the analogous [TMCl_*n*_(PNP)_2_] (where TM = transition metal and *n* = 1 or 2) core, revealed an inverse correlation between the P—TM—P bite angles and N⋯TM contact distances.

## Introduction

Nitro­gen-containing phosphines and phosphinites are ligands that have attracted significant attention in transition-metal coordination chemistry over the years (Hierso *et al.*, 2003 ▸; Cotton & Hong, 1992 ▸; Mayer & Kaska, 1994 ▸; Munzeiwa *et al.*, 2020 ▸). Amino­diphosphine derivatives, for instance, are versatile ligands, with the potential to be made more flexible by introducing alkyl chains (Blann *et al.*, 2005 ▸; Olding *et al.*, 2024 ▸) or more rigid by using fixed groups between the P atoms (Overett *et al.*, 2005 ▸; Beims *et al.*, 2023 ▸). Typically, amino­diphosphine ligands coordinate to metal centres *via* P-donor atoms, often excluding the N atom. However, the coordination behaviour and structural features of these ligands can be influenced by modifying the groups attached to the P or N atoms, enabling bridging or chelating coordination modes (Zhao *et al.*, 2018 ▸; Smith, 2022 ▸), and broadens their applications in coordination chemistry (Konrath *et al.*, 2019 ▸; Vasilenko *et al.*, 2016 ▸; Chirdon *et al.*, 2021 ▸).

Transition-metal com­plexes bearing [*M*Cl_*n*_(PNP)_2_] (*M* = Co, Ru, Cr or Mo; *n* = 1 or 2) cores in the literature seem to be understudied, especially considering structural features arising from ligand coordination, particularly ligand strain in the P—N—P backbone (Kim *et al.*, 2017 ▸; Fliedel *et al.*, 2016 ▸; Ogawa *et al.*, 2013 ▸; Aydemir *et al.*, 2011 ▸; Naicker *et al.*, 2022 ▸; Naktode *et al.*, 2014 ▸; Gaw *et al.*, 2000 ▸; Stennett *et al.*, 2012 ▸; Balakrishna *et al.*, 2003 ▸; Slawin *et al.*, 2004 ▸). While the existing literature predominantly focuses on synthesis (Slawin *et al.*, 2004 ▸), catalytic performance (Naicker *et al.*, 2022 ▸; Aydemir *et al.*, 2011 ▸; Ogawa *et al.*, 2013 ▸) and basic structural parameters (Gaw *et al.*, 2000 ▸; Fliedel *et al.*, 2016 ▸; Balakrishna *et al.*, 2003 ▸; Slawin *et al.*, 2004 ▸), the explicit effects of ligand-induced strain, particularly from small bite angles inherent in these amino­diphosphine ligands, remain inadequately explored. Notably, significant bite-angle contractions – reported as low as ∼69° in Ru^II^ com­plexes (Naicker *et al.*, 2022 ▸; Balakrishna *et al.*, 2003 ▸) and ∼71° in Co com­plexes (Fliedel *et al.*, 2016 ▸) – indicate the substantial strain imposed by these ligands, which likely influences both electronic and steric environments around the metal centres. However, only a few studies explicitly correlate these geometric constraints with broader chemical reactivity or stability implications (Ogawa *et al.*, 2013 ▸; Fliedel *et al.*, 2016 ▸). Moreover, the inter­play between such structural constraints and the van der Waals radii of central transition metals, which could further modulate metal–ligand inter­actions and catalytic outcomes, has rarely been addressed explicitly.

In this article, two new crystal structures of cobalt amino­diphosphine PNP com­plexes (**1** and **2**; Scheme 1) are reported featuring variation of the N-atom substituent, *i.e. n*-pentyl in com­plex **1** and isopropyl in com­plex **2**, allowing us to evaluate the effect of different N-atom substituents on the behaviour of the PNP ligand. A Cambridge Structural Database (CSD; Groom *et al.*, 2016 ▸) survey of com­plexes bearing [*M*Cl_*n*_(PNP)_2_] substructures was also used to gain insights into the correlation between metal–ligand geometric bond parameters and metal ion sizes. Finally, noncovalent inter­action (NCI) analysis was used to investigate the strain of the amino­diphosphine ligands along the P—N—P backbone caused by the various substituents and metal ions. With this in mind, investigation of ligand-strain effects and associated steric-electronic consequences in [*M*Cl_*n*_(PNP)_2_] com­plexes represents a significant research gap, promising to yield valuable insights into the structure–reactivity relationships critical for their application in catalysis and materials science (Fliedel *et al.*, 2016 ▸; Ogawa *et al.*, 2013 ▸; Naicker *et al.*, 2022 ▸).

## Experimental

### Materials and equipment

All experiments were performed using standard Schlenk techniques under inert conditions in moisture-free reaction glassware with anhydrous solvents. All solvents were of analytical grade. To render the reaction glassware moisture free, they were heated with a heat gun, followed by cycles of vacuum and nitro­gen purges. The solvents utilized were dry unless otherwise stated. Diethyl ether and hexane were distilled from sodium benzo­phenone under nitro­gen. Di­chloro­methane was distilled from P_2_O_5_ and ethanol from magnesium turnings. Complexes **1** and **2** were synthesized 
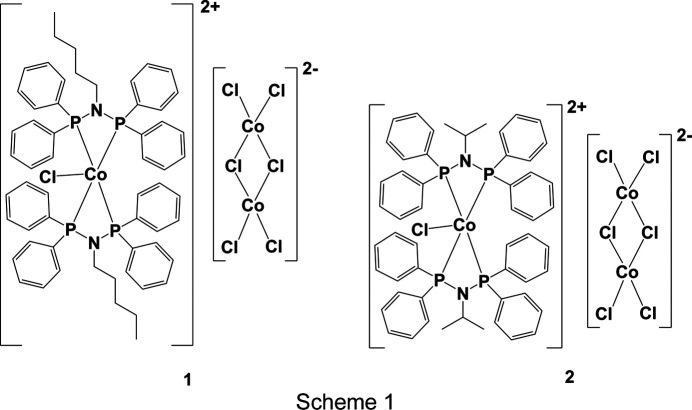
following the literature procedure of Naicker *et al.* (2015 ▸). Crystals of **1** and **2** were grown by the vapour diffusion of diethyl ether into a solution of the com­plexes in di­chloro­methane at room temperature to give blue crystals for **1** and **2**.

### Crystal structure analyses

Crystal data, data collection and structure refinement details are summarized in Table 1 ▸. All H atoms were positioned geometrically and allowed to ride on their respective parent atoms. All H atoms were refined isotropically. The disordered *n*-pentyl group was modelled with split occupancies, with the major com­ponent having a site-occupancy factor of 0.55 (3). In com­plex **2**, a solvent mask was calculated and 218 electrons were found in a volume of 830 Å^3^ in two voids per unit cell. This is consistent with the presence of 1 C_4_H_10_O and 0.25 CH_2_Cl_2_ molecules per asymmetric unit, which account for 42 and 10 electrons per unit cell, respectively.

### Noncovalent inter­actions (NCI) analysis

Noncovalent inter­action (NCI) calculations were per­for­med with *NCIPLOT4* (Boto *et al.*, 2020 ▸), a density-based tool that maps subtle inter­molecular forces. NCI analysis was carried out on the basis of promolecular approximations using the crystallographic atomic coordinates of the various metal complexes of interest. Reduced density gradient (RDG) isosurfaces were rendered in *VMD 1.9.3* (Humphrey *et al.*, 1996 ▸) and coloured according to sign(λ_2_)ρ(**r**), where ρ(**r**) is the electron density and λ_2_ is the second eigenvalue of the Hessian matrix. Deep-blue (large negative) values pinpoint strongly attractive regions, such as classical hy­dro­gen bonds, green zones near zero correspond to weak van der Waals contacts and red (positive) values flag steric repulsion (Boto *et al.*, 2020 ▸). The identical colour scale links the 3D isosurfaces (drawn for −0.07 ≤ isovalue ≤ 0.07) to the corresponding 2D NCI plots produced with *GNUPLOT 4.6* (Williams *et al.*, 2017 ▸). To gauge backbone strain, P—N—P atom sets were extracted from the CIFs of our transition-metal amino­diphosphine com­plexes (and analogous CSD entries) and analyzed in isolation. An analogous protocol, using the atomic coordinates of the *M*—P—N—P metallocycle, elucidated the character of the *M*⋯N contact within each com­plex.

## Results and discussion

### Description of X-ray crystal structures

**Figure 1:**
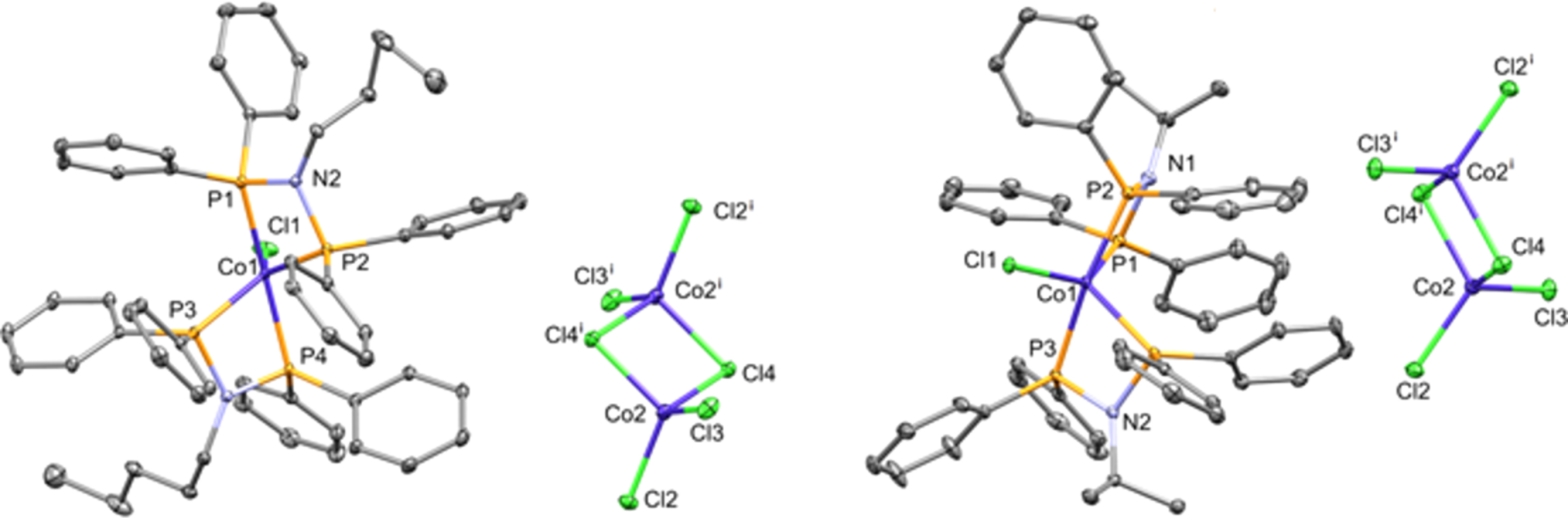
The mol­ecular structures of com­plexes **1** (left) and **2** (right), with displacement ellipsoids drawn at the 50% probability level. All H atoms and disordered *n*-pentyl (in **1**) and isopropyl (in **2**) groups have been omitted for clarity.

Blue crystals of **1** and **2** were obtained by vapour diffusion of diethyl ether into a di­chloro­methane solution of the respective com­plexes. The mol­ecular structures of com­plexes **1** and **2** are shown in Fig. 1 ▸. In both com­plexes, two ligands coordinate in a bidentate manner to the cobalt metal centres *via* the P atoms. Selected bond distances and angles are given in Table 2 ▸.

The asymmetric unit of com­plex **1** contains two species: the five-coordinated cationic com­plex [CoCl{κ^2^-*P*,*P*-(*N*)-C_5_H_11_}_2_]^2+^and the anionic dimer [Co_2_(μ_2_-Cl)_2_Cl_4_]^2−^. The cation has a distorted trigonal-bipyramidal geometry, with the Cl atom and one P atom from each PNP ligand in the equatorial plane, and the remaining two P atoms in axial positions. In contrast, the asymmetric unit of **2** contains a mol­ecule of the five-coordinated cation [CoCl{κ^2^-*P*,*P*-(*N*)-C_3_H_7_}_2_]^2+^ and half of the [Co_2_(μ_2_-Cl)_2_Cl_4_]^2−^ anion. The cationic species in **2** adopts a coordination geometry similar to that of **1**.

The equatorial planes of the two com­plexes show P—Co—P angles of 105.67 (2) and 109.47 (2)°, and Cl—Co—P angles of 131.45 (3) and 122.88 (2)° in com­plex **1**, and 135.914 (18) and 114.239 (17)° in com­plex **2**. These angles are indicative of a dis­torted trigonal-pyramidal geometry. Similar geometrical dis­­tor­tions have been reported in other penta­coordinated transition-metal com­plexes with diphosphine ligands (Nak­tode *et al.*, 2014 ▸; Fliedel *et al.*, 2016 ▸). This was further qu­anti­fied by calculating the φ (tau) index, defined as τ = α − β/60, where α and β correspond to two angles. The τ values for a perfect square-based pyramid and a perfect trigonal bipy­ra­mid are 0 and 1, respectively (Addison *et al.*, 1984 ▸). The structural distortion indices for **1** and **2** were calculated as 0.14 and 0.36, confirming the deviation from ideal geometries and the presence of inter­mediate distortion between square-pyramidal and trigonal-bipyramidal extremes.

The P—N—P bite angle of the bidentate ligand is acute, generating two P—N—P—Co metallacycle planes in each of the com­plexes, with inter­planar angles between 71.58 (3) and 105.28 (4)° in both com­plexes, and with the Co atom displaced slightly towards one of the P atoms. Complex **1** exhibits relatively uniform Co—P bond lengths, with equatorial bonds [2.2514 (6) and 2.2590 (6) Å] slightly shorter than the axial ones [2.2480 (6) and 2.2495 (6) Å]. In contrast, com­plex **2** shows greater asymmetry, with two longer Co—P distances [2.2913 (5) and 2.2654 (5) Å] and two shorter ones [2.2239 (6) and 2.2259 (6) Å], involving both equatorial and axial positions. The Co—Cl bond lengths are close in the two com­plexes [2.2305 (6) and 2.2398 (4) Å] and similar to the same distances in structures found in the literature.

### CSD survey

A com­prehensive survey of the Cambridge Structural Database (CSD, Version 5.40 with updates; Groom *et al.*, 2016 ▸) yielded 20 crystal structures of transition-metal com­plexes bearing bidentate amino­diphosphine (PNP) ligands with a [TMCl_*n*_(PNP)_2_] core (TM = transition metal and *n* = 1 or 2) (Table 3 ▸). Ruthenium was the most represented (with 8), followed by cobalt(II) (7), chromium (3) and molybdenum(II) (2). Notably, the ligands feature diverse substituents on the N atom, mainly alkyl or aryl groups, with occasional heteroatom-containing functionalities. Most com­plexes adopt an octa­hedral geometry (14 out of 20), while trigonal-bipyramidal geometries are less common, occurring in only six structures. This distribution reflects the combined steric and electronic influences of the PNP ligands and the inherent preferences of the metal centres.

**Figure 2:**
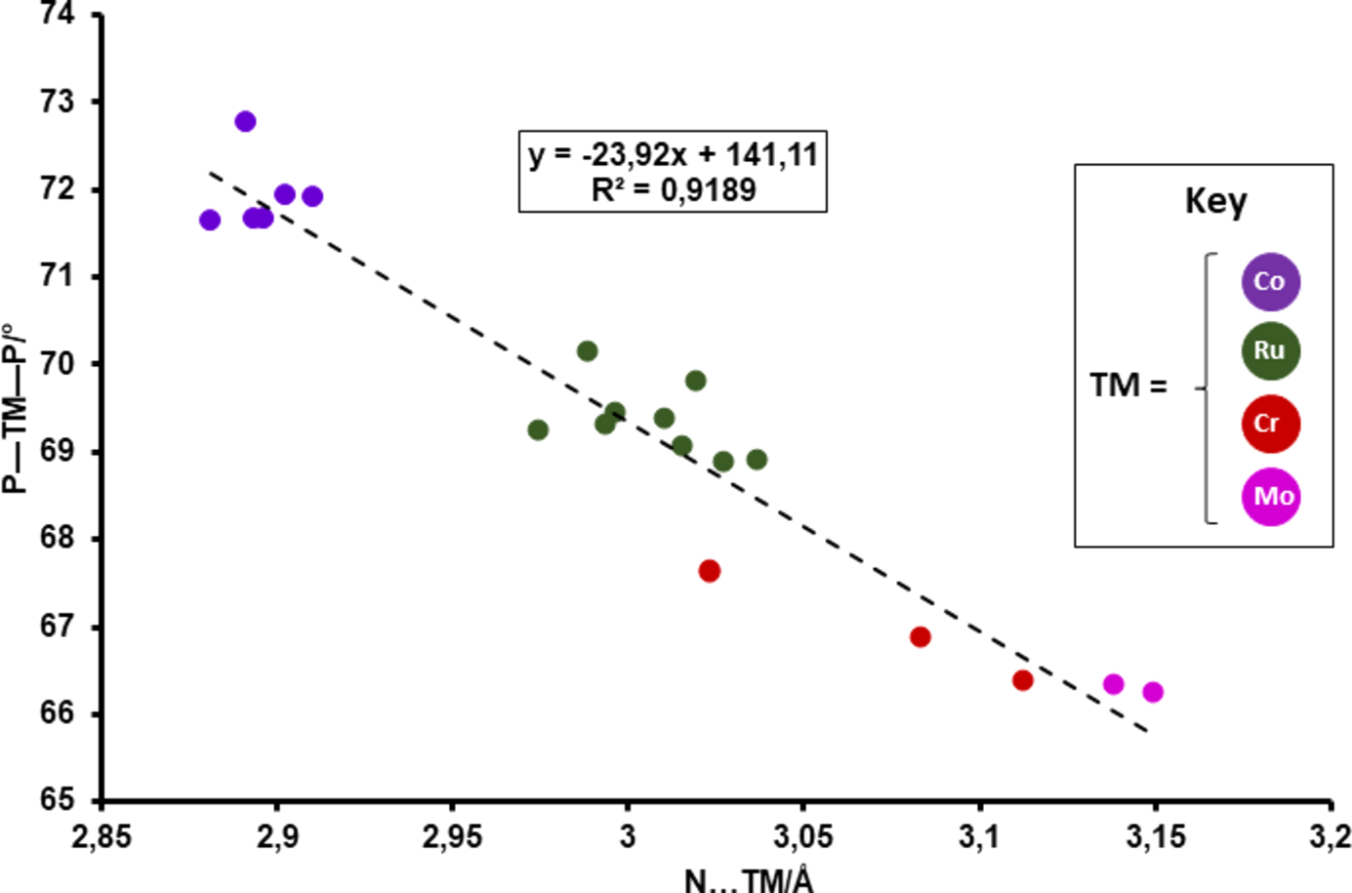
Correlation between the P—TM—P bite angles and N⋯TM contact distances in related PNP amino­diphosphine metal com­plexes found in the CSD.

One key geometric observation across the dataset is an inverse correlation between the P—TM—P bite angle and the nonbonded N⋯TM contact distance. As can be seen in the scatter plot (Fig. 2 ▸), com­plexes with larger P—TM—P bite angles exhibit shorter N⋯TM contacts. This suggests that wider bite angles may push the N atom into closer proximity with the metal centre, despite the lack of any formal bonding. The observed trend correlates well with the van der Waals radii of the metals: smaller metals like cobalt (1.63 Å) can accommodate wider P—TM—P angles, resulting in shorter N⋯TM distances. In contrast, larger metals such as ruthenium (1.78 Å) and molybdenum (1.90 Å) typically exhibit narrower bite angles and longer N⋯TM separations. Chromium, with an inter­mediate radius (1.66 Å), shows values consistent with this relationship.

The nature of the metal centre significantly influences the conformation of the PNP ligand (the P⋯P distance in particular). A CSD study of uncoordinated PNP gave an average P⋯P distance of 2.988 Å (Engelbrecht *et al.*, 2011 ▸; Keat *et al.*, 1981 ▸; Dunesha *et al.*, 2016 ▸; Cotton *et al.*, 1996 ▸; Gimbert *et al.*, 1999 ▸; Tobias & Hans-Christian, 2012 ▸; Luo *et al.*, 2013 ▸; Cloete *et al.*, 2009 ▸; Liu, 2014 ▸), showing flexibility and an unconstrained backbone. On coordination, a shorter P⋯P distance due to chelation was observed. This chelation effect is observed in com­plexes **1** and **2**, which have P⋯P distances of 2.6248 (8)–2.6263(9) and 2.5895 (6)–2.6528 (5) Å, respectively. Similar trends were observed across the 20 related transition-metal com­plexes from the CSD: Cr com­plexes showed P⋯P distances ranging between 2.723 and 2.755 Å, Mo between 2.712 and 2.729 Å, Ru between 2.63 and 2.679 Å, and Co between 2.616 and 2.697 Å (Table 3 ▸). The trend suggests that smaller metal centres, such as Co (van der Waals radius ≃ 1.63 Å), pull the P atoms closer together, leading to shorter P⋯P distances. In contrast, larger metals like Mo (≃1.90 Å) allow a more extended ligand conformation, resulting in longer P⋯P separations (Duncan Lyngdoh *et al.*, 2018 ▸). Additionally, P—TM bonding increases the electron-density redistribution, which strengthens the P—TM bonds and indirectly contributes to a shortening of the P⋯P distances (Rauch *et al.*, 2020 ▸). In complexes **1** and **2**, the steric properties of the N-substituent do not substantially alter the P⋯P separation [2.6248 (8)–2.6263 (9) Å in **1** and 2.5895 (6)–2.6528 (5) Å in **2**], even though they could still contribute to the overall ligand environment.

### NCI analysis of P⋯P and Co⋯N contacts

**Figure 3:**
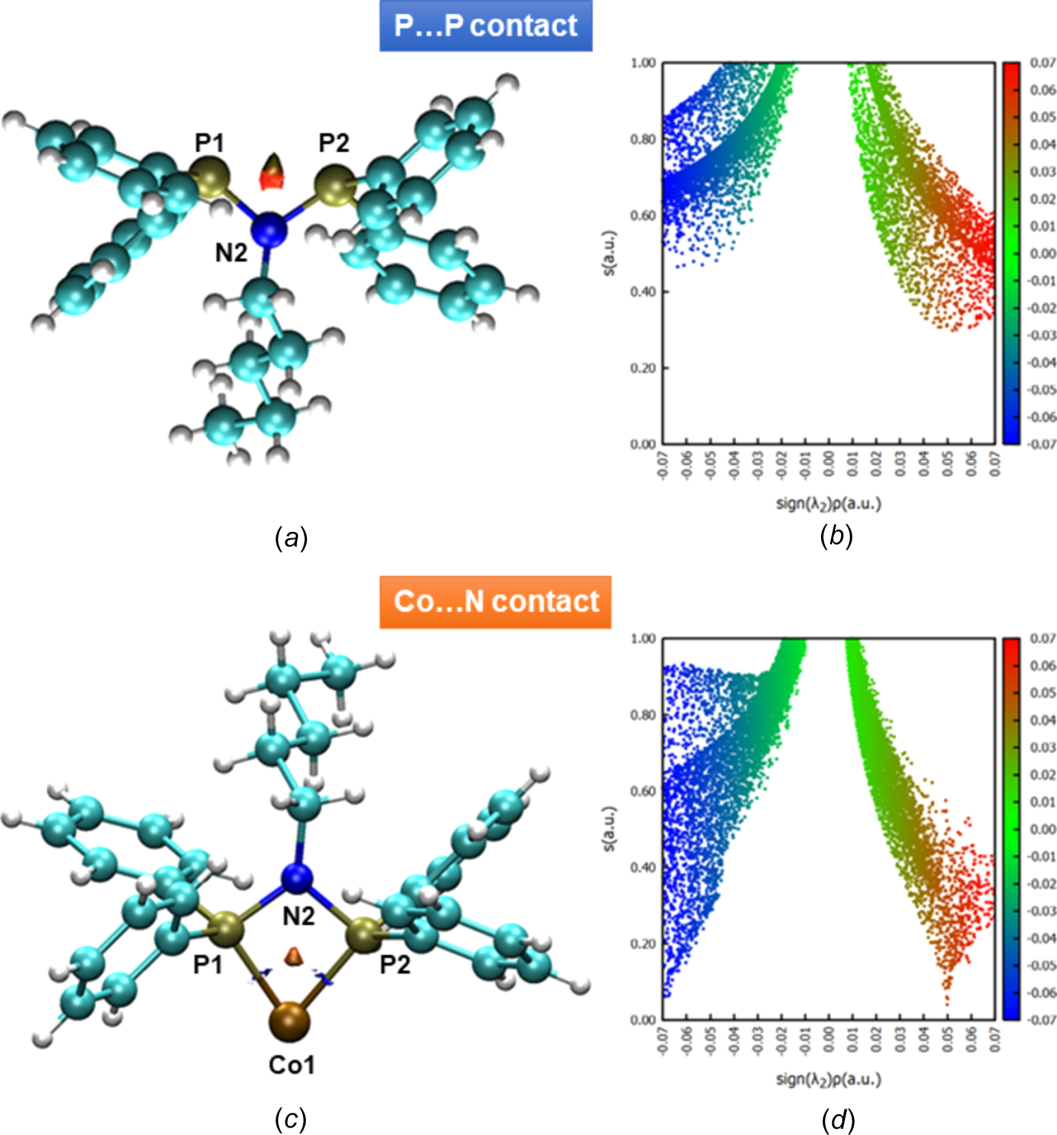
(*a*) 3D and (*b*) 2D representations of the NCI plots of the coordinated amino­diphosphine ligands in com­plex **1**. (*c*) 3D and (*d*) 2D representations of the NCI plots focusing on the Co⋯N contacts in com­plex **1**.

Noncovalent interaction (NCI) analysis was conducted using the promolecular approximation, which provides a computationally efficient and qualitative description of NCI regions, but should not be relied upon for quantitative electron-density analysis. Inter­actions were assessed using 3D NCI plots com­plemented by 2D scatter plots from reduced density gradient (RDG) calculations, which are shown in Fig. 3 ▸. For the P⋯P contacts, distinct regions were identified by orange-coloured RDG isosurfaces in the 3D representation [Fig. 3 ▸(*a*)]. This coloration indicates relatively strong steric repulsion or destabilizing inter­actions, consistent with close inter­atomic distances significantly shorter than the sum of the P-atom van der Waals radii (∼3.6 Å). These shortened distances suggest significant steric hindrance between the phospho­rus centres, emphasizing repulsive inter­actions rather than stabilizing dis­persive inter­actions. The 2D scatter plots support this inter­pretation, displaying characteristic peaks in the positive region of the electron density multiplied by the second Hessian eigenvalue, confirming the predominantly repulsive nature of these inter­actions.

The NCI analysis of the Co⋯N inter­action within com­plex **1** also showed orange-coloured RDG isosurfaces, indicating a notable degree of steric repulsion or strain around the metal coordination site. The Co⋯N contacts exhibited features consistent with partially destabilizing inter­actions, which likely arise due to steric constraints imposed by ligand architecture or coordination geometry. Correspondingly, the 2D scatter plots revealed distinctive peaks extending into the positive region of electron density multiplied by the second Hessian eigenvalue, further substanti­ating the sterically dominated nature of the Co⋯N inter­actions.

**Figure 4:**
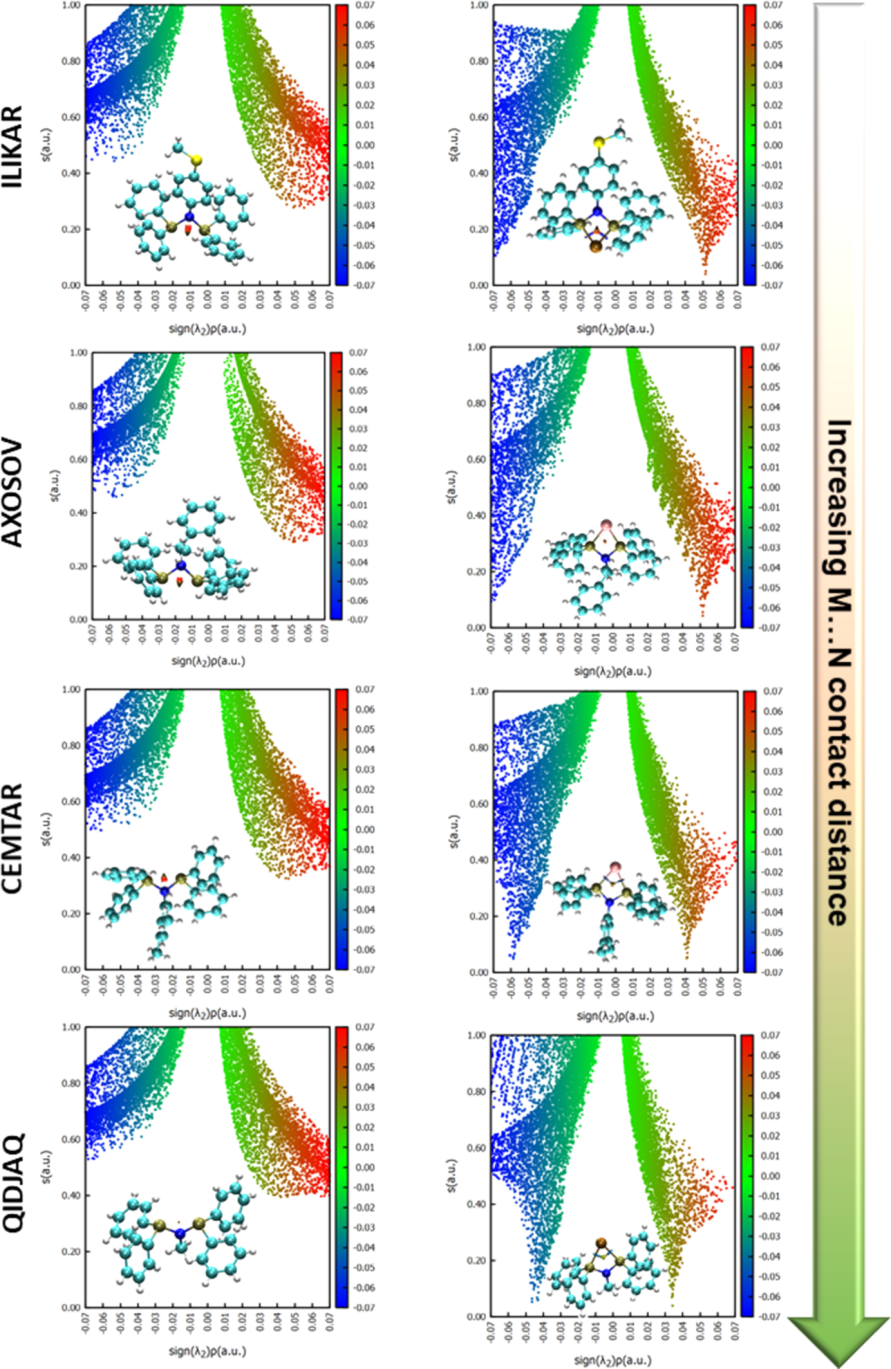
3D representations and corresponding 2D NCI plots for selected CSD refcodes with coordinated amino­diphosphine ligands (left) and their transition-metal com­plexes (right).

The NCI analysis was extended to other [TMCl_*n*_(PNP)_2_] com­plexes according to the CSD survey in Section 3.2. Only one of each representative transition-metal type of [TMCl_*n*_(PNP)_2_] com­plexes was selected for this analysis. The 3D representations and corresponding 2D scatter plots demonstrated pronounced orange-coloured isosurfaces and positive RDG peaks between the P atoms in the ligands (Fig. 4 ▸), similar to that of com­plex **1** [Figs. 3 ▸(*a*) and 3(*b*)], thus suggesting a repulsion rather than attractive dispersive inter­action between the P atoms. Upon coordination with Co (CSD refcode ILIKAR; Fliedel *et al.*, 2016 ▸) and Ru (AXOSOV; Díez *et al.*, 2004 ▸), similar orange-coloured RDG isosurfaces persist in the *M*⋯N inter­action zones, alongside characteristic positive peaks around the 0.05 sign(λ_2_)ρ(**r**) eigenvalue in the corresponding 2D scatter plot (Fig. 4 ▸). As for the Mo (CEMTAR; Ogawa *et al.*, 2013 ▸) and Cr (QIDJAQ; Stennett *et al.*, 2012 ▸) com­plexes, the *M*⋯N inter­action zones also exhibited characteristic positive peaks around the 0.04 and 0.035 sign(λ_2_)ρ(**r**) values, respectively, which could imply that there is a lesser degree of repulsion between the transition metal and its corresponding ligand. Inter­estingly, the sign(λ_2_)ρ values of the Mo—P bond in CEMTAR were found to be around −0.06, which is greater than that of Co—P (ILIKAR) and Ru—P (AXOSOV), which are ≤ −0.07. This implies that the Mo—P bond has an inherently weaker attraction between the Mo and P atoms than that seen for Co—P (ILIKAR) and Ru—P (AXOSOV). Finally, looking at the Cr—P bond in QIDJAQ, we observe sign(λ_2_)ρ(**r**) values between −0.05 and −0.04, which is typically in the range of hy­dro­gen bonding (Zamisa *et al.*, 2022 ▸) or weak noncovalent inter­actions (Mphahlele *et al.*, 2023 ▸). This suggests that the Cr—P bond in QIDJAQ is much weaker than the other *M*—P bonds investigated in this work and this could be the determining factor behind elongated Cr⋯N distances which ultimately leads to weaker coordination of the amino­diphosphine ligand to Cr.

## Conclusion

Two novel cobalt com­plexes (**1** and **2**), each bearing a [CoCl(PNP-κ^2^*P*,*P*′)_2_] core, were successfully synthesized and structurally characterized in the solid state. Both adopt distorted trigonal-bipyramidal geometries with coordination occurring exclusively through the P atoms of the PNP ligands. Complexes **1** and **2** feature five-coordinated [CoCl{κ^2^-*P*,*P*-(*N*)-C_5_H_11_}_2_]^2+^ and [CoCl{κ^2^-*P*,*P*-(*N*)-C_3_H_7_}_2_]^2+^ cationic spe­cies, respectively, along with [Co_2_(μ_2_-Cl)_2_Cl_4_]^2−^ counter-ions. A com­prehensive CSD survey, com­plemented by noncovalent inter­action (NCI) analysis, provided further insight into the relationship between structural features and metal identity. The survey confirmed that octa­hedral geometries dominate among [TMCl_*n*_(PNP)_2_] com­plexes, while trigonal-bipyramidal geometries are relatively rare, reflecting the steric and electronic constraints imposed by the PNP ligand framework and the preferences of individual metal centres. A key trend identified was an inverse correlation between the P—TM—P bite angles and the N⋯TM contact distances, which is largely governed by the van der Waals radii of the metals. Smaller metals like cobalt (1.63 Å) accommodate wider bite angles and shorter stronger N⋯TM inter­actions. In contrast, larger metals such as ruthenium (1.78 Å) and molybdenum (1.90 Å) exhibit narrower bite angles and longer N⋯TM contacts, with chromium showing inter­mediate behaviour. NCI analysis revealed notable steric repulsion at Co⋯N contact points, indicating strain within the coordination sphere likely induced by the constrained ligand geometry. Similar effects were observed across the broader dataset of [TMCl_*n*_(PNP)_2_] com­plexes. The sign(λ_2_)ρ eigenvalues further suggest that Mo—P bonds are weaker than their Co—P and Ru—P counterparts, with Cr—P inter­actions being the weakest overall. Despite the potential for moderate-to-strong *M*⋯N inter­actions, steric hindrance from the ligand backbone significantly limits such coordination. However, to obtain a more accurate representation of the electron-density distribution, NCI analysis based on quantum-chemical wavefunction is required, as it accounts for electronic relaxation, polarization and charge-transfer effects that the promolecular approximation cannot capture. These insights highlight how subtle structural and electronic factors — such as ligand bite angle, metal size and steric effects — govern coordination in PNP-ligated transition-metal com­plexes, offering valuable guidance for their design in catalysis and materials chemistry.

## Supplementary Material

Crystal structure: contains datablock(s) 1, 2, global. DOI: 10.1107/S2053229625009519/ef3070sup1.cif

CCDC references: 2499418, 2499419

## Figures and Tables

**Table 1:** Experimental details Experiments were carried out at 100 K with Mo *K*α radiation using a Bruker SMART APEXII area-detector diffractometer. Absorption was corrected for by multi-scan methods (*SADABS*; Bruker, 2009 ▸). H-atom parameters were constrained.

	**1**	**2**
Crystal data		
Chemical formula	[CoCl(C_29_H_31_NP_2_)_2_][Co_2_Cl_6_]	[CoCl(C_27_H_27_NP_2_)_2_][Co_2_Cl_6_]
*M*_r_	1336.01	1279.90
Crystal system, space group	Triclinic, *P* 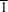	Monoclinic, *P*2_1_/*c*
*a*, *b*, *c* (Å)	11.5114 (4), 12.6404 (4), 20.7858 (7)	12.2179 (2), 15.4508 (2), 30.4455 (5)
α, β, γ (°)	89.481 (2), 82.999 (2), 68.324 (2)	90, 97.070 (1), 90
*V* (Å^3^)	2787.57 (17)	5703.69 (15)
*Z*	2	4
μ (mm^−1^)	1.38	1.34
Crystal size (mm)	0.35 × 0.22 × 0.14	0.40 × 0.18 × 0.14

Data collection		
*T*_min_, *T*_max_	0.640, 0.746	0.672, 0.746
No. of measured, independent and observed [*I* ≥ 2σ(*I*)] reflections	74041, 13808, 11199	131209, 14218, 11950
*R*_int_	0.042	0.029
(sin θ/λ)_max_ (Å^−1^)	0.667	0.669

Refinement		
*R*[*F*^2^ > 2σ(*F*^2^)], *wR*(*F*^2^), *S*	0.040, 0.113, 1.06	0.030, 0.077, 1.05
No. of reflections	13808	14218
No. of parameters	691	629
No. of restraints	242	96
Δρ_max_, Δρ_min_ (e Å^−3^)	1.13, −0.63	0.50, −0.35

Computer programs: *COSMO* (Bruker, 2009 ▸), *SAINT-Plus* (Bruker, 2009 ▸), *SHELXS* (Sheldrick, 2008 ▸), *olex2.refine* (Bourhis *et al.*, 2015 ▸), *OLEX2* (Dolomanov *et al.*, 2009 ▸) and *PLATON* (Spek, 2020 ▸).

**Table 2:** Selected bond lengths (Å) and angles (°) in the solid-state structures of com­plexes **1** and **2**

	**1**	**2**
Bond lengths		
Co1—P1	2.2495 (6)	2.2239 (6)
Co1—P2	2.2514 (6)	2.2259 (6)
Co1—P3	2.2590 (6)	2.2654 (5)
Co1—P4	2.2480 (6)	2.2913 (5)
Co1—Cl1	2.2305 (6)	2.2400 (5)
Co2—Cl2	2.2469 (7)	2.2315 (6)
Co2—Cl3	2.2292 (7)	2.2274 (6)
Co2—Cl4	2.3412 (7)	2.3398 (6)
		
Bond angles		
P1—Co1—P2	71.35 (2)	71.16 (2)
P1—Co1—P3	103.72 (2)	101.61 (2)
P2—Co1—P3	105.67 (2)	170.58 (2)
P4—Co1—P1	170.63 (2)	109.47 (2)
P4—Co1—P2	101.97 (2)	105.03 (2)
P4—Co1—P3	71.28 (2)	71.194 (19)
P1—Co1—Cl1	95.27 (2)	135.91 (2)
P2—Co1—Cl1	131.45 (3)	91.89 (2)
P1—N1—P2	–	99.14 (9)
P1—N2—P2	101.71 (9)	–
P3—N1—P4	101.34 (9)	–
P3—N2—P4	–	102.23 (9)

**Table 3:** CSD survey of transition-metal com­plexes of the type [TMCl_*n*_(PNP)_2_] (*n* = 1 or 2) TBP is trigonal bipyramidal and OCT is octa­hedral.

CSD refcode	Geometry	Dihedral (°)	N⋯TM (Å)	P—N—P (°)	P⋯P (Å)	P—TM—P (°)	Reference
AXOSOV	TBP	77.952	2.993	100.222	2.63	69.325	Díez *et al.* (2004 ▸)
CEMTAR	OCT	4.052	3.149	103.215	2.712	66.275	Ogawa *et al.* (2013 ▸)
CEMTEV	OCT	5.091	3.138	104.698	2.729	66.354	Ogawa *et al.* (2013 ▸)
DOSWIT	OCT	1.229	2.891	103.565	2.697	72.79	Naktode *et al.* (2014 ▸)
EFARIP	OCT	8.797	3.083	105.642	2.723	66.894	Kim *et al.* (2017 ▸)
FEHZOK	OCT	6.272	2.893	102.604	2.652	71.669	Fliedel *et al.* (2016 ▸)
FEHZUQ	OCT	3.505	2.902	102.157	2.659	71.949	Fliedel *et al.* (2016 ▸)
ILIJIY	TBP	67.528	2.896	102.652	2.653	71.688	Fliedel *et al.* (2016 ▸)
ILIJOE	TBP	75.979	2.881	101.681	2.622	71.654	Fliedel *et al.* (2016 ▸)
ILIKAR	TBP	74.301	2.91	99.429	2.616	71.927	Fliedel *et al.* (2016 ▸)
FOQGAT	OCT	0	3.019	101.187	2.679	69.828	Gaw *et al.* (2000 ▸)
HUWLIU	OCT	4.387	2.974	102.386	2.641	69.256	Balakrishna *et al.* (2003 ▸)
HUWLOA	OCT	0	3.027	101.48	2.662	68.908	Balakrishna *et al.* (2003 ▸)
QAMJAQ	OCT	0	3.015	101.783	2.662	69.096	Slawin *et al.* (2004 ▸)
QIDJAQ	OCT	1.703	3.023	108.93	2.755	67.635	Stennett *et al.* (2012 ▸)
QIDJAQ	OCT	1.331	3.023	109.334	2.75	67.661	Stennett *et al.* (2012 ▸)
SESBAX	OCT	0	3.036	101.165	2.666	68.923	Naicker *et al.* (2022 ▸)
UMERUA	TBP	81.557	2.988	100.194	2.648	70.18	Aydemir *et al.* (2011 ▸)
UMESAH	OCT	0	3.01	101.518	2.663	69.392	Aydemir *et al.* (2011 ▸)
XEFXAI	TBP	66.281	3.112	107.16	2.747	66.392	Jabri *et al.* (2006 ▸)
PEHHIT	OCT	4.94	2.996	101.611	2.655	69.468	Lu *et al.* (1993 ▸)

## Data Availability

The authors declare no competing interests.

## References

[bb1] Addison, A. W., Rao, T. N., Reedijk, J., van Rijn, J. & Verschoor, G. C. (1984). *J. Chem. Soc. Dalton Trans.* pp. 1349–1356.

[bb2] Aydemir, M., Baysal, A., Özkar, S. & Yıldırım, L. T. (2011). *Inorg. Chim. Acta* **367**, 166–172.

[bb3] Balakrishna, M. S., Panda, R. & Mague, J. T. (2003). *Polyhedron* **22**, 587–593.

[bb4] Beims, N., Greven, T., Schmidtmann, M. & van der Vlugt, J. I. (2023). *Chem. A Eur. J.* **29**, e202302463. 10.1002/chem.202302463 37873907

[bb5] Blann, K., Bollmann, A., Dixon, J. T., Hess, F. M., Killian, E., Maumela, H., Morgan, D. H., Neveling, A., Otto, S. & Overett, M. J. (2005). *Chem. Commun.* pp. 620–621. 10.1039/b412431f 15672155

[bb6] Boto, R. A., Peccati, F., Laplaza, R., Quan, C., Carbone, A., Piquemal, J.-P., Maday, Y. & Contreras-Garcı, A. J. (2020). *J. Chem. Theory Comput.* **16**, 4150–4158. 10.1021/acs.jctc.0c00063 32470306

[bb7] Bourhis, L. J., Dolomanov, O. V., Gildea, R. J., Howard, J. A. K. & Puschmann, H. (2015). *Acta Cryst.* A**71**, 59–75. 10.1107/S2053273314022207 PMC4283469 25537389

[bb8] Bruker (2009). *COSMO*, *SAINT-Plus* and *SADABS*. Bruker AXS Inc., Madison, Wisconsin, USA.

[bb9] Chirdon, D. N., Kelley, S. P., Hazari, N. & Bernskoetter, W. H. (2021). *Organometallics* **40**, 4066–4076.

[bb10] Cloete, N., Visser, H. G., Roodt, A. & Gabrielli, W. F. (2009). *Acta Cryst.* E**65**, o3081. 10.1107/S1600536809045978 PMC2972116 21578811

[bb11] Cotton, A. F., Kühn, F. E. & Yokochi, A. (1996). *Inorg. Chim. Acta* **252**, 251–256.

[bb12] Cotton, F. A. & Hong, B. (1992). *Prog. Inorg. Chem* **40**, 179.

[bb13] Díez, J., Gamasa, M. P., Gimeno, J., Rodríguez, Y. & García–Granda, S. (2004). *Eur. J. Inorg. Chem.* **2004**, 2078–2085.

[bb14] Dolomanov, O. V., Bourhis, L. J., Gildea, R. J., Howard, J. A. K. & Puschmann, H. (2009). *J. Appl. Cryst.* **42**, 339–341.

[bb15] Duncan Lyngdoh, R. H., Schaefer, H. F. III & King, R. B. (2018). *Chem. Rev.* **118**, 11626–11706. 10.1021/acs.chemrev.8b00297 30543419

[bb16] Dunesha, N., Pramod, B. P. & Holger, B. F. (2016). *Z. Kristallogr. New Cryst. Struct.* **231**, 653–656.

[bb17] Engelbrecht, I., Visser, H. G. & Roodt, A. (2011). *Acta Cryst.* E**67**, o2041–o2042. 10.1107/S1600536811027656 PMC3213490 22091069

[bb18] Fliedel, C., Rosa, V., Vileno, B., Parizel, N., Choua, S., Gourlaouen, C., Rosa, P., Turek, P. & Braunstein, P. (2016). *Inorg. Chem.* **55**, 4183–4198. 10.1021/acs.inorgchem.5b02889 27054464

[bb19] Gaw, K. G., Smith, M. B. & Slawin, A. M. Z. (2000). *New J. Chem.* **24**, 429–435.

[bb20] Gimbert, Y., Robert, F., Durif, A., Averbuch, M.-T., Kann, N. & Greene, A. E. (1999). *J. Org. Chem.* **64**, 3492–3497. 10.1021/jo982245o 11674471

[bb21] Groom, C. R., Bruno, I. J., Lightfoot, M. P. & Ward, S. C. (2016). *Struct. Sci.* **72**, 171–179. 10.1107/S2052520616003954 PMC4822653 27048719

[bb22] Hierso, J.-C., Amardeil, R., Bentabet, E., Broussier, R., Gautheron, B., Meunier, P. & Kalck, P. (2003). *Coord. Chem. Rev.* **236**, 143–206.

[bb23] Humphrey, W., Dalke, A. & Schulten, K. (1996). *J. Mol. Graph.* **14**, 33–38. 10.1016/0263-7855(96)00018-5 8744570

[bb24] Jabri, A., Crewdson, P., Gambarotta, S., Korobkov, I. & Duchateau, R. (2006). *Organometallics* **25**, 715–718.

[bb25] Keat, R., Manojlović-Muir, L., Muir, K. W. & Rycroft, D. S. (1981). *J. Chem. Soc. Dalton Trans.* pp. 2192–2198.

[bb26] Kim, E. H., Lee, H. M., Jeong, M. S., Ryu, J. Y., Lee, J. & Lee, B. Y. (2017). *ACS Omega* **2**, 765–773. 10.1021/acsomega.6b00506 PMC6641062 31457469

[bb27] Konrath, R., Spannenberg, A. & Kamer, P. (2019). *Chem. A Eur. J.* **25**, 15341–15350. 10.1002/chem.201903379 PMC6916561 31495988

[bb28] Liu, X.-F. (2014). *Inorg. Chim. Acta* **421**, 10–17.

[bb29] Lu, Y. Z. Z.-Z., Zhao, W.-J., Wang, H.-G. & Ma, Y.-F. (1993). *Jiegou Huaxue (Chin.) (Chin. J. Struct. Chem.)* **12**, 129.

[bb30] Luo, L.-J., Liu, X.-F. & Gao, H.-Q. (2013). *J. Coord. Chem.* **66**, 1077–1083.

[bb31] Mayer, H. A. & Kaska, W. C. (1994). *Chem. Rev.* **94**, 1239–1272.

[bb32] Mphahlele, M. J., El-Gogary, T. M. & Zamisa, S. J. (2023). *J. Mol. Struct.* **1294**, 136501.

[bb33] Munzeiwa, W. A., Omondi, B. & Nyamori, V. O. (2020). *Beilstein J. Org. Chem.* **16**, 362–383. 10.3762/bjoc.16.35 PMC7082614 32256853

[bb34] Naicker, D., Alapour, S., Zamisa, S. J. & Friedrich, H. B. (2022). *J. Coord. Chem.* **75**, 1129–1146.

[bb35] Naicker, D., Friedrich, H. B. & Omondi, B. (2015). *RSC Adv.* **5**, 63123–63129.

[bb36] Naktode, K., Kottalanka, R. K., Adimulam, H. & Panda, T. K. (2014). *J. Coord. Chem.* **67**, 3042–3053.

[bb37] Ogawa, T., Kajita, Y., Wasada-Tsutsui, Y., Wasada, H. & Masuda, H. (2013). *Inorg. Chem.* **52**, 182–195. 10.1021/ic301577a 23231761

[bb38] Olding, A., Lucas, N. T., Ho, C. C. & Bissember, A. C. (2024). *Dalton Trans.* **53**, 4471–4478. 10.1039/d3dt04269c 38348688

[bb39] Overett, M. J., Blann, K., Bollmann, A., Dixon, J. T., Hess, F., Killian, E., Maumela, H., Morgan, D. H., Neveling, A. & Otto, S. (2005). *Chem. Commun.* pp. 622–624. 10.1039/b412432d 15672156

[bb40] Rauch, M., Kar, S., Kumar, A., Avram, L., Shimon, L. J. W. & Milstein, D. (2020). *J. Am. Chem. Soc.* **142**, 14513–14521. 10.1021/jacs.0c05500 PMC7453403 32786799

[bb41] Sheldrick, G. M. (2008). *Acta Cryst.* A**64**, 112–122. 10.1107/S0108767307043930 18156677

[bb42] Slawin, A. M. Z., Milton, H. L., Wheatley, J. & Woollins, J. D. (2004). *Polyhedron* **23**, 3125–3132.

[bb43] Smith, M. B. (2022). *Molecules* **27**, 6293.

[bb44] Spek, A. L. (2020). *Acta Cryst.* E**76**, 1–11. 10.1107/S2056989019016244 PMC6944088 31921444

[bb45] Stennett, T. E., Haddow, M. F. & Wass, D. F. (2012). *Organometallics* **31**, 6960–6965.

[bb46] Tobias, M. & Hans-Christian, B. (2012). *Z. Naturforsch. B* **67**, 504–506.

[bb48] Vasilenko, V., Roth, T., Blasius, C. K., Intorp, S. N., Wadepohl, H. & Gade, L. H. (2016). *Beilstein J. Org. Chem.* **12**, 846–853. 10.3762/bjoc.12.83 PMC4901889 27340475

[bb49] Williams, T., Kelley, C., Bröker, H., Campbell, J., Cunningham, R., Denholm, D., Elber, E., Fearick, R., Grammes, C. & Hart, L. (2017). *GNUPLOT 4.6*. http://www.gnuplot.info/.

[bb50] Zamisa, S. J., Ngubane, N. P., Adeleke, A. A., Jonnalagadda, S. B. & Omondi, B. (2022). *Cryst. Growth Des.* **22**, 5814–5834.

[bb51] Zhao, P.-H., Ma, Z.-Y., Hu, M.-Y., He, J., Wang, Y.-Z., Jing, X.-B., Chen, H.-Y., Wang, Z. & Li, Y.-L. (2018). *Organometallics* **37**, 1280–1290.

